# Unusual location of apocrine hidrocystoma in children: Case series

**DOI:** 10.1016/j.ijscr.2023.108419

**Published:** 2023-06-22

**Authors:** Carmine Noviello, Mercedes Romano, Letizia Trotta, Roberto Alfano, Andrea Ronchi, Alfonso Papparella

**Affiliations:** a Pediatric Surgery Unit, Department of Woman, Child, General and Specialized Surgery, University of Campania “Luigi Vanvitelli”, Naples, Italy; b Department of Advanced Medical and Surgical Sciences “DAMSS”, University of Campania “Luigi Vanvitelli”, Naples, Italy; c Pathology Unit, Department of Mental and Physical Health and Preventive Medicine, University of Campania “Luigi Vanvitelli”, Naples, Italy

**Keywords:** Apocrine hidrocystoma, Sweat gland, Children

## Abstract

**Introduction and importance:**

Apocrine Hidrocystoma is a relatively rare benign tumour that begins from the apocrine sweat glands of the head and neck. The Authors present a case series of children with urogenital localization.

**Cases presentation:**

Two boys (15 years and 9 years) presented with a small mass on the glans. Another 15-year-old boy presented with a cystic lesion in the right side of the scrotum where he had a previous surgery. The last case, a 17-year-old boy, presented because of a penile cyst of 8 mm. All four had surgical operations because of aesthetic discomfort or problems during micturition. Histological examination showed a diagnosis of apocrine hidrocystoma in all cases.

**Clinical discussion:**

This benign tumour rarely affects the urogenital system in children, but when it happens the child can have discomfort and proper treatment is mandatory.

**Conclusion:**

Surgery is the preferred treatment with a low risk of recurrence.

## Highlights

• Apocrine hidrocystoma is quite rare, benign tumour, beginning from the apocrine sweat glands of the head and neck.
• Rarely this tumour involves the uro-genital system.
• Is is a rare tumour in the uro-genital system of children.
• We present 4 cases who required surgery because of aesthetic discomfort and problem during micturition.

## 1 Background

Apocrine Hidrocystoma (AH) is a relatively rare benign tumour that begins with the apocrine sweat glands [1]. The most affected site is the upper body (head and neck), but it can occur everywhere else in the body where these glands are present (axilla, external auditory canal, eyelids and nipple) [2]. It primarily affects adults, does not have a sexual predilection and is uncommon in children [3]. It usually presents with an intradermal solitary nodule, dome-shaped, translucent, blue-black, with a diameter between 3 mm and 15 mm [4]. Clinically, it is generally asymptomatic, but presenting as swelling, can lead to problems on the site where it is located [5]. Normally it is presented in a single form, multiple forms are rare. No clear predisposing factors are reported [6]. The final diagnosis can be established by a histological examination. The authors present their experience with case series in male children, which resulted in localization problems in the urogenital system.

## 2 Methods

This is a case report from our pediatric surgical department, and has been reported in accordance with the PROCESS criteria [7].

### 2.1 Case 1 presentation

**Fig. 1:**
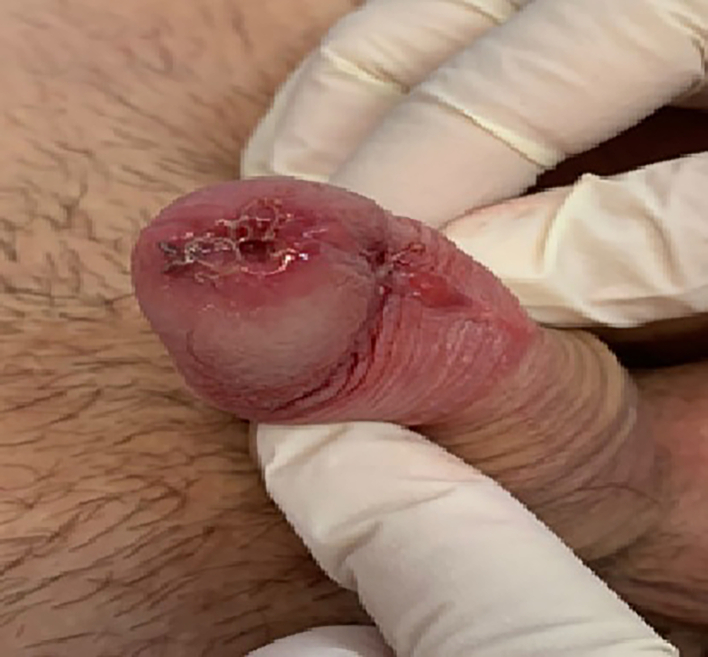
Post-operative urethral reconstruction following removal of the lesion.

**Fig. 2:**
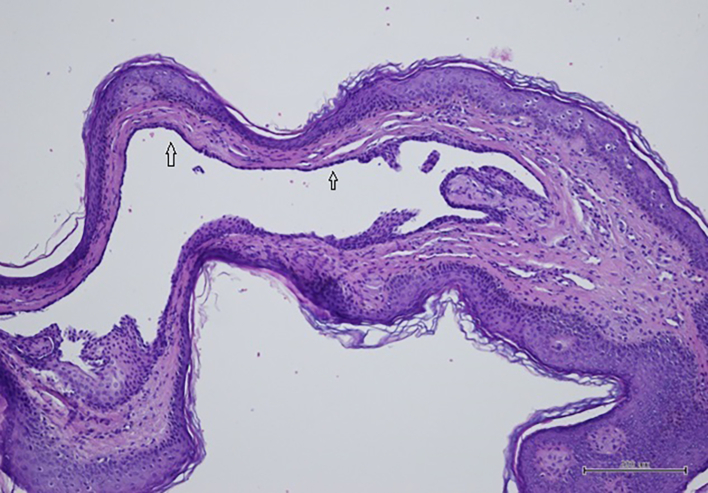
Histological examination of apocrine hidrocystoma showing a unilocular apocrine epithelial-lined cyst (arrows) in the dermis (hematoxylin and eosin, original magnification 100×).

A 15-year-old boy came to our attention reporting a small painless mass on the glans appeared about a year and with a growing trend. The boy complained for several months of aesthetic discomfort and problems while urinating. The personal and family history was not relevant. Clinically, the patient exhibited cystic swelling (approximately 1 cm in diameter) on the glans gland just next and inside the urethral meatus. As a result of reported problems, surgical excision with sedation and local anaesthesia was performed. During surgery, the lesion appeared larger: extending along the urethra to approximately 2 cm. After complete excision, the urethral meatus was reconstructed (Fig. 1) and a urethral stent was left in place for 48 h. At follow-up of 6 months and 1 year the aesthetic result was good and the boy did not complain of urinary problems. Histological examination showed a cystic lesion delimited by a single cubic epithelium with focal presence of Citokeratin7 (CK7) cells and a peripheral layer of myoepithelial cells (p63 positive): apocrine hidrocystoma (Fig. 2).

### 2.2 Case 2 presentation

**Fig. 3:**
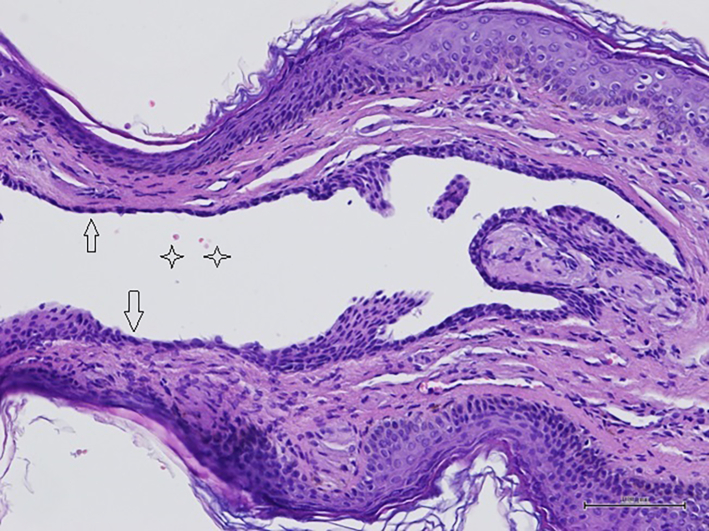
Histological examination of apocrine hidrocystoma: the cyst is lined by two cell layers epithelium (arrows), with focal papillary projections and evidence of classic decapitation secretion (stars) (hematoxylin and eosin, original magnification 200×).

A 9-year-old child came to our hospital for the presence of a cyst approximately 3 mm at the base of the glans, in the ventral region. A further check was carried out at the end of 6 months: the cyst had slightly increased in volume. The child reported discomfort while retracting the foreskin and was recommended for surgery. The surgery was performed with sedation and local anaesthesia. The size of the excised lesion was 4 mm and was limited to the superficial portion of the glans without affecting the urethra. At one-year follow-up, the lesion was not present and the patient was doing well. Histological examination showed a cystic lesion delimited by a single cubic epithelium and a peripheral layer of myoepithelial cells: hidrocystoma apocrine (Fig. 3).

### 2.3 Case 3 presentation

**Fig. 4:**
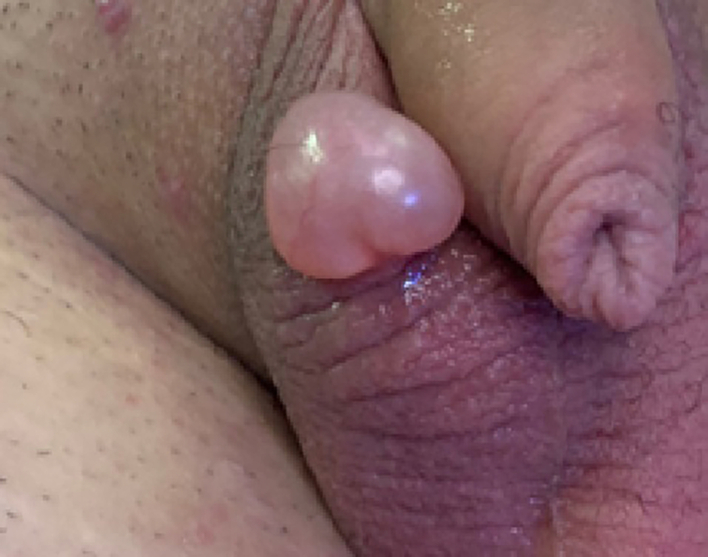
Apocrine hidrocystoma of the scrotum.

A 15-year-old boy presented with a cystic lesion in right side of the scrotum. This patient had undergone transcrotal orchidopexy 1 year earlier in another hospital, a month after the surgery he had a dehiscence of the wound which was sutured. Six months later, a cystic lesion appears on the scrotal scar. No pain was reported, just discomfort while wearing clothes. The lesion looked full of liquid, translucent and measured 3 cm in diameter (Fig. 4). An ultrasound was performed to determine any testicular involvement. The surgery was performed with sedation and local anaesthesia. The cyst was dissected through the normal skin of the scrotum and completely removed. The one-year follow-up was good, free of dehiscence and cystic recurrence. Histology showed the same features as the above cases.

### 2.4 Case 4 presentation

A 17-year-old boy came to our attention because of a localized penis pain during erection. During the clinical examination, the patient showed a recurvatum of the glans, a hypertrophy of the frenulum and an 8 mm cystic formation on the penis. The boy complained of pain in the area where the cyst was and increased size. Therefore, the surgical procedure was performed with total removal of the cyst in sedation and local anaesthesia. Histologic analysis led to the diagnosis of Hidrocystoma apocrine. During the six-month follow-up, no pain or cyst was present.

## 3 Discussion

Apocrine hidrocystoma (AH) is a rare benign proliferation of the sweat glands present in the head and neck [8]. It can rarely affect other areas such as the oral mucosa, ears, trunk and genitalia [5]. It usually occurs as a single injury, but can be multiple, and may be a sign of rare pathology: Schopf-Schulz-Passarge syndrome or Goltz-Gorlin syndrome [9,10]. These hereditary disorders, also known as ectodermic dysplasia, show developmental anomalies with formation of cysts in two or more of the following structures: hair, teeth, nails, sweat glands and other related ectodermal structures derived. The aetiology is unknown. Histopathologically, this tumour results from the secretory part of the apocrine sweat glands where a cystic proliferation of these glands occurs, rather than a (instead of a) simple cystic retention of secretion. Histological characteristics are typical: an inner wall coated with single or double layers of cuboidal-columnar epithelium without secretion, a peripheral layer of flat myoepithelial cells and the presence of lipofuscin granules. With respect to the pediatric age and atypical sites, not much is reported in the scientific literature: only two cases of localization in the scrotum [11,12] and two in the glans [5,13]. Sometimes, the cystic formations of the glans are misdiagnosed and described as parameatal cysts or in rare cases create diagnostic problems with serious pathologies of the penis [14]. The localized forms to the foreskin [15,16] and the penis [[17], [18], [19]] are best described, but not among children. In all cases reported in the literature, there is no prior trauma, although in our second case there was a previous surgical procedure with complication at the site of the wound. Pain and discomfort are caused by the presence of swelling in a highly sensitive area such as the genitals. With regard to the diagnosis, it is primarily clinical, but with histological confirmation, however in the forms of skin involvement may play a role new techniques of artificial intelliince, as happens for melanoma [20]. No spontaneous involution of AH is recorded. Treatment can be surgical (complete excision of the lesion) or conservative (needle puncture after topical application of 1 % atropine cream or scopolamine creams, alternatively laser vaporization with carbon dioxide), but in the first case less recurrence is reported [21,22].

## 4 Conclusion

The diagnosis of AH remains clinical and histological. The tumour is benign, but when it affects particular districts, such as genitalia, it can cause problems. The treatment can be surgical or conservative, but in the second case the recurrence seems more common. Based on our experience in childhood, we can say that surgical removal is the preferred treatment for easy surgierremoval and the lower risk of recurrence.

## Informed consent

Written informed consent was obtained from the patient's parents/legal guardian for publication and any accompanying images. A copy of the written consent is available for review by the Editor-in-Chief of this journal on request.

## Ethical approval

Our University (University of Campania “Luigi Vanvitelli”) exempts the case reports from ethical approval because it is a small group of patients and does not involve any experimental procedures.

## Funding

No funding was received.

## Author contribution

Noviello Carmine: Study conception and design, Drafting of the manuscript

Trotta Letizia: Data acquisition

Romano Mercedes: Analysis and data interpretation

Alfano Roberto: data analysis

Ronchi Andrea: histology analisys

Papparella Alfonso: Critical revision.

## Guarantor

Noviello Carmine.

## Research registration number

N/A.

## Declaration of competing interest

The authors declare that there is no conflict of interests.

## References

[1] Mehregan A.H. (1964). Apocrine cystadenoma; a clinicopathologic study with special reference to the pigmented variety. Arch. Dermatol..

[2] Kikuchi K., Fukunaga S., Inoue H., Miyazaki Y., Ide F., Kusama K. (2014). Apocrine hidrocystoma of the lower lip: a case report and literature review. Head Neck Pathol..

[3] López V., Alonso V., Jordá E., Santonja N. (2013). Apocrine hidrocystoma on the penis of a 40-year-old man. Int. J. Dermatol..

[4] Smith J.D., Chernosky M.E. (1974). Apocrine hidrocystoma (cystadenoma). Arch. Dermatol..

[5] Samplaski M.K., Somani N., Palmer J.S. (2009). Apocrine hidrocystoma on glans penis of a child. Urology.

[6] Jo J.W., Yang J.W., Jeong D.S. (Aug 2019). Apocrine hidrocystoma on the penis: report of a case and review of the previous cases. Ann. Dermatol..

[7] Agha R.A., Sohrabi C., Mathew G., Franchi T., Kerwan A., O’Neill N for the PROCESS Group (2020). The PROCESS 2020 guideline: updating consensus preferred reporting of case series in surgery (PROCESS) guidelines. Int. J. Surg..

[8] Sarabi K., Khachemoune A. (Sep 6 2006). Hidrocystomas - a brief review. Medscape Gen. Med..

[9] Ascherman J.A., Knowles S.L., Troutman K.C. (Jul 2002). Extensive facial clefting in a patient with Goltz syndrome: multidisciplinary treatment of a previously unreported association. Cleft Palate Craniofac. J..

[10] Gira A.K., Robertson D., Swerlick R.A. (Feb 2004). Multiple eyelid cysts with palmoplantar hyperkeratosis—quiz case. Arch. Dermatol..

[11] Flessati P., Camoglio F.N., Bianchi S., Fasoli L., Menghi A. (Jan-Feb 1999). An apocrine hidrocystoma of the scrotum. A case report. Minerva Chir..

[12] Park J., Kim I., Jang H.C., Chae I.S., Park K., Kim Y., Chung H. (Jul 2015). Linear skin-coloured papules on scrotum: a quiz. Apocrine hidrocystoma. Acta Derm. Venereol..

[13] Taylor D., Juhl M.E., Krunic A.L., Sidiropoulos M., Gerami P. (Mar 2015). Apocrine hidrocystoma of the urethral meatus: a case report. Acta Derm. Venereol..

[14] Bani-Hani M., Nawafleh S., Al-Zubi M. (2020). Penile calciphylaxis diagnosis and treatment challenges a case report. Int. J. Surg. Case Rep..

[15] Shao I.H., Chen T.D., Shao H.T., Chen H.W. (Sep 14 2012). Male median raphe cysts: serial retrospective analysis and histopathological classification. Diagn. Pathol..

[16] Ahmed A., Jones A.W., Apocrine cystadenoma. (Dec 1969). A report of two cases occurring on the prepuce. Br. J. Dermatol..

[17] de Dulanto F., Armijo-Moreno M., Camacho Martinez F. (1973). Hidradénome nodulaire (cystadénome apocrine) du pénis [Nodular hidradenoma (apocrine cystadenoma) of the penis]. Ann. Dermatol. Syphiligr. (Paris).

[18] Powell R.F., Palmer C.H., Smith E.B. (Sep 1977). Apocrine cystadenoma of the penile shaft. Arch. Dermatol..

[19] Mataix J., Bañuls J., Blanes M., Pastor N., Betlloch I. (Sep 2006). Translucent nodular lesion of the penis. Apocrine hidrocystoma of the penis. Arch. Dermatol..

[20] Taher H., Grasso V., Tawfik S., Gumbs A. (2022). The challenges of deep learning in artificial intelligence and autonomous actions in surgery: a literature review. Art. Int. Surg..

[21] Lambert W.C., Wiener B.D., Schwartz R.A., Quillen C.G., Giampapa V.C. (1984). The giant apocrine hidrocystoma. J. Surg. Oncol..

[22] Gupta S., Handa U., Handa S., Mohan H. (Apr 2001). The efficacy of electrosurgery and excision in treating patients with multiple apocrine hidrocystomas. Dermatol. Surg..

